# No evidence for the involvement of the argasid tick *Ornithodoros faini* in the enzootic maintenance of marburgvirus within Egyptian rousette bats *Rousettus aegyptiacus*

**DOI:** 10.1186/s13071-016-1390-z

**Published:** 2016-03-05

**Authors:** Amy J. Schuh, Brian R. Amman, Dmitry A. Apanaskevich, Tara K. Sealy, Stuart T. Nichol, Jonathan S. Towner

**Affiliations:** Viral Special Pathogens Branch, Division of High-Consequence Pathogens and Pathology, Centers for Disease Control and Prevention, Atlanta, GA USA; United States National Tick Collection, the James H. Oliver, Jr. Institute for Coastal Plain Science, Georgia Southern University, Statesboro, GA USA

**Keywords:** Filovirus, Marburgvirus, Marburg virus, Tick, Argasid, *Ornithodoros*, Egyptian rousette bat, *Rousettus aegyptiacus*, Transmission, Maintenance

## Abstract

**Background:**

The cave-dwelling Egyptian rousette bat (ERB; *Rousettus aegyptiacus*) was recently identified as a natural reservoir host of marburgviruses. However, the mechanisms of transmission for the enzootic maintenance of marburgviruses within ERBs are unclear. Previous ecological investigations of large ERB colonies inhabiting Python Cave and Kitaka Mine, Uganda revealed that argasid ticks (*Ornithodoros faini*) are hematophagous ectoparasites of ERBs. Yet, their potential role as transmission vectors for marburgvirus has not been sufficiently assessed.

**Findings:**

In the present study, 3,125 *O. faini* were collected during April 2013 from the rock crevices of Python Cave, Uganda. None of the ticks tested positive for marburgvirus-specific RNA by Q-RT-PCR. The probability of failure to detect marburgvirus at a conservative prevalence of 0.1 % was 0.05.

**Conclusions:**

The absence of marburgvirus RNA in *O. faini* suggests they do not play a significant role in the transmission and enzootic maintenance of marburgvirus within their natural reservoir host.

## Findings

### Introduction

The genus *Marburgvirus* (Filoviridae), includes a single species, *Marburg marburgvirus*, with two virus members, Marburg virus (MARV) and Ravn virus (RAVV) (hereafter collectively referred to as marburgvirus). These viruses cause outbreaks of hemorrhagic disease perpetuated by human-to-human transmission, with reported case fatality ratios up to 90 %. Ecological investigations have identified the cave-dwelling Egyptian rousette bat (ERB; *Rousettus aegyptiacus*) as a natural reservoir host for marburgvirus [1, 2], based upon the detection of marburgvirus RNA and IgG antibodies [3, 4] and the isolation of infectious marburgvirus [1–3, 5] from ERBs inhabiting caves associated with recent human outbreaks. Experimental infection of captive ERBs with MARV has confirmed their natural reservoir host status by demonstrating MARV replication in a range of tissues [6–8] and oral shedding of infectious virus in the absence of clinical disease [7, 8] followed by seroconversion [6–8]. Although it has been hypothesized that MARV is transmitted between ERBs by direct contact with infectious MARV oral secretions through activities such as touching, licking and biting [7], a recent study was unable to show horizontal transmission within a 42 day period from MARV-experimentally infected ERBs to susceptible in-contact ERBs [9]. It has been suggested that enzootic transmission and maintenance of marburgvirus within the ERB population may involve an intermediate host, such as an arthropod vector [1, 7–9].

Argasid ticks (*Ornithodoros faini*; = *Carios faini, Alectorobius faini*) have been observed on the bodies of ERBs inhabiting a cave in Kruger National Park, South Africa [10] and previous ecological investigations of large ERB colonies inhabiting Python Cave and Kitaka Mine, Uganda revealed that *O. faini* are hematophagous ectoparasites of ERBs [1, 2]. *Ornithodoros* spp. ticks are known vectors of several arboviruses including African swine fever [11], bluetongue [12], Karshi [13], Langat [13, 14] and Qalyub [15] viruses. A previous collection of approximately 300 adult and nymphal argasid ticks taken from rock crevices near ERB roosting sites at Python Cave and Kitaka Mine were negative for marburgvirus RNA by Q-RT-PCR [1, 2]. However, given the limited sample size, further collection and testing of these arthropods was considered important to determine whether they play a role in the enzootic transmission and maintenance of marburgvirus.

## Methods

A total of 3,125 adult and nymph argasid ticks were individually collected using forceps from small rock crevices near bat roosting sites within Python Cave, Queen Elizabeth National Park, Uganda in April 2013. The tick collections were undertaken with the approval of the Uganda Wildlife Authority and performed in accordance with a protocol approved by the Centers for Disease Control and Prevention’s Institutional Animal Care and Use Committee. The cave is inhabited by a sole chiropteran population consisting of approximately 40,000 ERB individuals with a consistent 2.5 % prevalence of active marburgvirus infection [2]. Between 2007 and 2008, two epidemiologically unrelated cases of Marburg hemorrhagic fever occurred in tourists 7–10 days after visiting Python Cave [16, 17].

Pools of five ticks were placed directly in 2-mL grinding vials (OPS Diagnostics, Lebanon, NJ) containing 250 μL of a 1:1 ratio of MagMax Lysis Binding Solution (Life Technologies, Grand Island, NY) to isopropanol (MagMax Lysis Binding buffer). The tick pools were homogenized for 2 min at 1,500 strokes per minute using the GenoGrinder 2000 (OPS Diagnostics, Lebanon, NJ). After the addition of 550 μL of MagMax Lysis buffer, the pools were transferred to cryovials and immediately stored under liquid nitrogen vapors. Nucleic acid was extracted using the MagMax Pathogen RNA/DNA Kit (Life Technologies, Grand Island, NY) on the MagMax Express-96 Deep Well Magnetic Particle Processor (Life Technologies, Grand Island, NY). All samples were analyzed by quantitative-reverse transcriptase-polymerase chain reaction (Q-RT-PCR) on the 7500 Real-Time PCR System (Life Technologies, Grand Island, NY) using the SuperScript III Platinum One-Step Q-RT-PCR Kit (Life Technologies, Grand Island, NY) with marburgvirus-specific primers and probes targeting the viral protein 40 gene [2], as well as with tick-specific primer and probes targeting the mitochondrial 16 s ribosomal RNA (rRNA) gene (endogenous control to confirm nucleic acid integrity).

A short region (~450 bp) of the 16 s rRNA gene of three samples was amplified using the SuperScript III One-Step RT-PCR System with the Platinum Taq High Fidelity DNA Polymerase Kit (Life Technologies, Grand Island, NY) and then sequenced using the Big Dye Terminator v3.1 Cycle Sequencing Kit (Life Technologies, Grand Island, NY) and six primers on the ABI Prism 3100 Genetic Analyzer (Life Technologies, Grand Island, NY). These sequences [GenBank: KU295468- KU295470], as well as morphological examination of a set of ticks preserved in 70 % ethanol, were used to confirm the *O. faini* species designation.

## Results and discussion

None of the tick pools (0/625) were positive for marburgvirus-specific RNA, while 95.7 % (598/625) of the pools were positive for tick-specific 16srRNA (4.3 % of the pools were 16 s rRNA negative indicating the presence of nucleic acid inhibitors in these samples). The probability of failure to detect marburgvirus RNA in this sample size of 2,990 ticks (598 pools of 5) at a conservative prevalence of 0.1 % was 0.05.

Adult *Ornithodoros* spp. feed and reproduce repeatedly, [18] and survive up to 20 years [19]. Further, these ticks have been shown to harbor infectious African swine fever virus for more than five years [20], transmit Langat virus more than three years after oral exposure [13] and transmit Karshi virus nearly eight years following oral exposure [21]. The natural history of *Ornithodoros* spp. suggests that if *O. faini* was a vector for marburgvirus then its presence would have been detected in our tick collection. However, an experimental infection study showed that ERBs inoculated with a large dose of MARV (4 log_10_TCID_50_) exhibited relatively low peak viremias (3.3 log_10_TCID_50_ genome equivalents/mL) of short duration (3 days) [7]. Furthermore, an ecological investigation revealed that only 5 % of wild-caught ERBs shown to be actively infected with marburgvirus had detectable viremias [2]. Together these data suggest that marburgvirus-infected ERBs may not produce sufficiently high viremias for feeding *O. faini* to acquire the virus. Similarly, the low viremias would make mechanical transmission unlikely. It is also possible that either the midgut epithelial or salivary gland cells of *O. faini* are refractory to marburgvirus infection or replication. A previous laboratory study found that *Ornithodoros* spp. ticks intrathoracically inoculated with Reston virus (Family Filoviridae), to bypass the midgut barrier, showed no evidence of virus replication [22].

In conclusion, this study found no evidence for *O. faini* playing a significant role in the enzootic transmission and maintenance of marburgvirus.

## Abbreviations

ERB: Egyptian fruit bat
MARV: Marburg virus
Q-RT-PCR: Quantitative-reverse transcriptase-polymerase chain reaction
rRNA: Ribosomal RNA
